# RNA-Seq Revealed Differences in Transcriptomes between 3ADON and 15ADON Populations of *Fusarium graminearum In Vitro* and *In Planta*

**DOI:** 10.1371/journal.pone.0163803

**Published:** 2016-10-27

**Authors:** Krishna D. Puri, Changhui Yan, Yueqiang Leng, Shaobin Zhong

**Affiliations:** 1 Department of Plant Pathology, North Dakota State University, Fargo, ND, United States of America; 2 Department of Computer Science, North Dakota State University, Fargo, ND, United States of America; University of Wisconsin Madison, UNITED STATES

## Abstract

*Fusarium graminearum* is the major causal agent of Fusarium head blight (FHB) in barley and wheat in North America. The fungus not only causes yield loss of the crops but also produces harmful trichothecene mycotoxins [Deoxynivalenol (DON) and its derivatives-3-acetyldeoxynivalenol (3ADON) and 15-acetyldeoxynivalenol (15ADON), and nivalenol (NIV)] that contaminate grains. Previous studies showed a dramatic increase of 3ADON-producing isolates with higher aggressiveness and DON production than the 15ADON-producing isolates in North America. However, the genetic and molecular basis of differences between the two types of isolates is unclear. In this study, we compared transcriptomes of the 3ADON and 15ADON isolates *in vitro* (in culture media) and *in planta* (during infection on the susceptible wheat cultivar ‘Briggs’) using RNA-sequencing. The *in vitro* gene expression comparison identified 479 up-regulated and 801 down-regulated genes in the 3ADON isolates; the up-regulated genes were mainly involved in C-compound and carbohydrate metabolism (18.6%), polysaccharide metabolism (7.7%) or were of unknown functions (57.6%). The *in planta* gene expression analysis revealed that 185, 89, and 62 genes were up-regulated in the 3ADON population at 48, 96, and 144 hours after inoculation (HAI), respectively. The up-regulated genes were significantly enriched in functions for cellular import, C-compound and carbohydrate metabolism, allantoin and allantoate transport at 48 HAI, for detoxification and virulence at 96 HAI, and for metabolism of acetic acid derivatives, detoxification, and cellular import at 144 HAI. Comparative analyses of *in planta* versus *in vitro* gene expression further revealed 2,159, 1,981 and 2,095 genes up-regulated in the 3ADON isolates, and 2,415, 2,059 and 1,777 genes up-regulated in the 15ADON isolates at the three time points after inoculation. Collectively, our data provides a foundation for further understanding of molecular mechanisms involved in aggressiveness and DON production of the two chemotype isolates of *F*. *graminearum*.

## Introduction

*Fusarium graminearum* is the major causal agent of Fusarium head blight (FHB) in North America and other regions of the world. The pathogen not only causes direct yield losses, but also produces various types of trichothecene mycotoxins [Deoxynivalenol (DON) and its acetylated forms (3-acetyl-4-deoxynivalenol = 3ADON and 15-acetyl-4-deoxvevalenol = 15ADON), nivalenol (NIV) and its acetylated form 4-acetylnevalenol] [1]. DON not only acts as a virulence factor during disease development [2, 3] but also poses severe health hazards to both human and animals [4–6]. FHB and DON are primarily managed through an integrated approach that combines use of moderately resistant cultivars and a timely fungicide application [7]. However, sources of effective FHB resistance are limited and use of a single source of resistance such as Sumai 3 in commercial cultivars may create selection pressure on the pathogen and lead to an outbreak of more virulent/aggressive pathogen population [8, 9].

In recent years, population genetics, global species structure and trichothecene chemotype diversity of the FHB pathogen complex have been extensively studied [10–13]. These studies indicate that one chemotype of the pathogen is dominant in specific geographic regions although other types may coexist in small fractions. Dominance of NIV-type isolates along with the low frequency of 15ADON- or 3ADON-type isolates is more common in Asian regions [14–16]. In North America, dominance of 15ADON-type isolates along with the presence of 3ADON- or NIV-type isolates was observed [17–20]. However, recent studies indicated that 3ADON-type isolates were significantly increased in Canada [10], North Dakota [18], and China [21] in recent years. The newly emerging 3ADON-type population appears to be more aggressive based on growth rate, disease severity on different cultivars with varied levels of resistance, and DON production *in vitro* [10, 18]. Our data from two years of field experiments using susceptible and moderately resistant wheat cultivars indicated that the 3ADON producers accumulated a higher level of DON on grains irrespective of host resistance after spray inoculation (Ali and Zhong, unpublished data). The recovery of *Fusarium* isolates from artificially inoculated heads with a mixture of both 3ADON- and 15ADON-type isolates indicated that the recovery frequencies were similar for both types of isolates (Ali and Zhong, unpublished data), suggesting that the 3ADON-type isolates do not have the advantage of outcompeting 15ADON-type isolates during infection. However, it is still not known why the 3ADON-type population produces a higher level of DON than the 15ADON-type population.

Trichothecenes produced by *F*. *graminearum* can be broadly categorized into two groups (type A or type B trichothecenes) based on presence or absence of oxygen atoms at carbon atoms 7 (C-7) and 8 (C-8) [1, 22]. Type A trichothecenes (T-2 toxin, HT-2 toxin, and 4, 15-diacetoxyscirpenol) lack the hydroxyl group at C-7 but have a hydroxyl group and ester group or no oxygen substitution at carbon atom C-8, whereas Type B trichothecenes (DON, NIV, and their derivatives) have a hydroxyl group at C-7 and a keto (carbonyl) group at C-8 [23–25]. DON and NIV have structural differences with the former lacking, and the latter possessing an oxygen atom at carbon C-4 [22, 26].

Trichothecene biosynthesis in *Fusarium* involves a complex pathway consisting of oxygenation, isomerization and esterification steps [22]. The enzymes involved in these biosynthetic steps are encoded by 15 *TRI* genes located at three loci, including the *TRI* core cluster with 12 genes (*Tri3*, *Tri4*, *Tri5*, *Tri6*, *Tri7*, *Tri8*, *Tri9*, *Tri10*, *Tri11*, *Tri12*, *Tri13*, and *Tri14*) [27], the two-gene (*Tri1-Tri16*) locus[28–30], and a single gene locus (*Tri101*) [31]. Whether DON or NIV is produced depends on the function of two genes, *Tri13* (cytochrome P450 monooxygenase) and *Tri7* (acetyltransferase) [32]. NIV producers contain functional *Tri13* and *Tri7* while DON producers carry non-functional copies of these two genes due to multiple deletion or insertion events [32, 33]. The genetic basis of 3ADON and 15ADON production and their biological significance are still not clear. Recently, Alexander et al. [34] indicated that *Tri8* (trichothecene C-3 esterase) regulates the production of 3ADON or 15ADON, and is required to convert the diacetylated 3- and 15- ADON intermediate into 3ADON and 15ADON, respectively. However, comparative studies of transcriptomes of the two trichothecene types (3ADON and 15ADON) during host infection have not been conducted and the molecular mechanisms involved in higher DON production and more aggressive nature of 3ADON isolates are not known.

Gene expression profiles and their relationships with virulence or aggressiveness and DON accumulation during *F*. *graminearum*-wheat and -barley interactions have been studied using DNA microarrays [35–39]. With the development of next generation sequencing technologies, new tools such as RNA-sequencing provide more effective approaches to study the gene expression profile changes of organisms under different conditions [40, 41]. The RNA-seq method is more sensitive than microarrays especially in detecting those transcripts that are rarely expressed [41]. Wang et al. [41] showed that 8 million reads were sufficient to reach RNA-Seq saturation for most samples with large genome sizes. Bashir et al. [42] have demonstrated that more than 90% of the transcripts in human samples can be adequately covered with just a million sequence reads. The coverage is more than sufficient to reach the saturation needed in RNA-Seq for small genomes such as those of filamentous fungi, while costs are comparable to the DNA microarray approach. Walkowiak et al. [40] used RNA-seq to compare gene expression profiles of one 3ADON strain and one 15ADON strain during interaction in cultures and identified genes expressed differentially between isolates and during their interaction. The overall goal of our study was to understand the molecular mechanisms that make 3ADON- and 15ADON-type populations different during infection on a susceptible cultivar with the following specific objectives: i) to compare the transcriptomes of the 3ADON- and 15ADON-type populations *in vitro* and *in planta* using the RNA-seq approach, and ii) to identify the expression differences of candidate genes related to aggressiveness and DON production between the two types of *F*. *graminearum* isolates.

## Materials and Methods

### Fungal isolates and wheat materials

*F*. *graminearum* isolates were collected during 2008 to 2010 from North Dakota (45° 56′—49° 00′ N, 96° 33′-104° 03′ W) and genotyped with trichothecene type specific primers [18]. Ten 3ADON-type isolates and ten 15ADON-type isolates (Table 1) were randomly selected and further characterized for disease aggressiveness and DON accumulation on three spring wheat genotypes [Grandin (susceptible), Steele-ND (moderately resistant), and ND 2710 (resistant)] in greenhouse experiments using the point inoculation method [43]. The plant growth conditions, inoculum preparation, inoculation, and disease scoring were the same as previously described [18]. For DON content measurement, grains from inoculated heads of each wheat genotype were harvested at maturity, manually threshed, ground to a fine powder using a coffee grinder, and then sent to the Veterinary Diagnostic Laboratory, North Dakota State University, for mycotoxin analyses.

**Table 1:** Information on 20 *Fusarium graminearum* isolates used in the study.

Isolate	Origin	Cultivar	Year	Chemotype	Collected by
**Fg 08–001**	Foster, ND, USA	Reeder	2008	3ADON	S. Zhong
**Fg 08–003**	Steele, ND, USA	wheat	2008	3ADON	S. Zhong
**Fg 08–004**	Barnes, ND, USA	wheat	2008	3ADON	S. Zhong
**Fg 08–005**	Foster, ND, USA	Reeder	2008	3ADON	S. Zhong
**Fg 08–006**	Steele, ND, USA	wheat	2008	3ADON	S. Zhong
**Fg 08–009**	Foster, ND, USA	Steele ND	2008	3ADON	S. Zhong
**Fg 08–010**	Barnes, ND, USA	wheat	2008	3ADON	S. Zhong
**Fg 08–012**	Steele, ND, USA	wheat	2008	3ADON	S. Zhong
**Fg 08–025**	ND, USA	wheat	2008	3ADON	S. Zhong
**Fg 08–029**	ND, USA	wheat	2008	3ADON	S. Zhong
**Fg 08–007**	Foster, ND, USA	Vantage	2008	15ADON	S. Zhong
**Fg 08–013**	Steele, ND, USA	wheat	2008	15ADON	S. Zhong
**Fg 08–026**	ND, USA	wheat	2008	15ADON	S. Zhong
**Fg 08–030**	ND, USA	wheat	2008	15ADON	S. Zhong
**Fg 08–034**	ND, USA	wheat	2008	15ADON	S. Zhong
**Fg 08–036**	ND, USA	wheat	2008	15ADON	S. Zhong
**Fg 08–037**	Foster, ND, USA	wheat	2008	15ADON	S. Zhong
**Fg 08–043**	Foster, ND, USA	Durum	2008	15ADON	S. Zhong
**Fg 08–057**	Foster, ND, USA	Alsen	2008	15ADON	S. Zhong
**09-1-H1-1**	Dicky, ND, USA	wheat	2009	15ADON	S. Ali

### Sample collection for RNA extraction

To collect the *in vitro* samples, spores from each of the ten 3ADON- or 15ADON-type isolates (Table 1) were harvested separately from axenic cultures on Mung Bean Agar (MBA), equally mixed for each type, and plated on the cellophane membranes overlaid on MBA media. The cultures were grown at 23±1°C with alternate 12 h dark and light cycles. At the fifth day after plating, mycelia were scraped, frozen immediately in liquid nitrogen, ground to a fine powder and stored at -80°C until use for RNA isolation. Two replicates were used for each type of isolates (3ADON-type and 15ADON-type) and thus, a total of four *in vitro* samples [two fungal populations (15ADON-type and 3ADON-type), two replicates, and one time point (five days after plating)] were obtained for RNA isolation.

The *in planta* samples were collected from the FHB susceptible wheat cultivar ‘Briggs’ [44] at three different time points after inoculation with the 3ADON- and 15ADON-type populations, respectively. Briefly, seven seeds of Briggs were planted in each of the 15-cm plastic pots filled with Sunshine pot mix (Sun Gro Horticulture Canada Ltd.) and maintained in a greenhouse with 16 h supplemental lights at 23±1°C. Plants were fertilized with slow releasing Osmocote^+^ (15:9:12) (Everris NA, Inc, Marysville OH) and Plantex 20-20-20 (Plant Products Co. Ltd, Ontario, Canada) at a two-week interval. At anthesis, eight to ten heads/pot were tagged and inoculated with a mixture of spores from ten isolates of the same type (Table 1) using the point inoculation method with 1000 spores per spikelet. The spikelets nearby to the inoculated one for each spike were marked with sharpie markers. After incubation in humidity chambers (misting run for 30 s on every 8 m to maintain 100% humidity) for 48 h at 26–27°C and with 18 h lights, the plants were returned to the greenhouse under normal conditions. The inoculated spikelets were collected from 8–10 heads per replicate at 48, 96 and 144 hours after inoculation (HAI), respectively, frozen immediately in liquid nitrogen, and stored at -80°C until use. For each type of isolates, two replicates were used. Thus, a total of 12 *in planta* samples [two fungal populations (15ADON-type and 3ADON-type), two replicates, and three time points (48, 96, and 144 HAI)] were obtained for RNA extraction.

### RNA extraction, library preparation, and sequencing

Total RNA was extracted from approximately 30 mg of each *in vitro* sample (mycelia) or *in planta* sample (plant and fungal tissues) (ground to a fine powder) using the SV total RNA isolation system (Promega BioSciences LLC, CA, USA) following the manufacturer’s instruction. The RNA samples were checked for quality using a 1.2% agarose gel, quantified by the Bio-Analyzer 2100 (Agilent Technologies, San Diego, CA), diluted to a concentration at 50 ng/μl and stored at -80°C until further use. Approximately 3μg of total RNA from each sample was used to prepare library using the TruSeq RNA sample preparation kit (Illumina, San Diego, CA) according to the manufacturer’s protocol. Briefly, the poly-A containing mRNA was purified from the total RNA using the poly-T oligo attached to the magnetic beads. After purification, mRNA was fragmented into small pieces using divalent cations under elevated temperature. The fragmented mRNA was converted to first strand cDNA using reverse transcriptase and random primers. Single strand cDNA was further converted to double strand (ds) cDNA using DNA polymerase I and RNase H. Then, the ds cDNA fragments were end repaired, ligated with indexing adapters, purified and enriched with PCR to develop the library. The prepared libraries were sent to Huntsman Cancer Institute, University of Utah (Salt Lake City, UT) for generating 50bp single-end reads with the Illumina HiSeq 2000 sequencing system.

### Mapping sequence reads to the reference genome and identification of differentially expressed genes (DEGs)

Mapping of sequence reads to reference genome, and analyses of transcript abundance and differential gene expression were performed as described by Trapnell et al. [45]. All sequence reads were trimmed to remove the low-quality sequences (the first 13 bases). The trimmed reads (37 bases) were then aligned to the *F*. *graminearum* reference genome downloaded from the Broad Institute (http://broadinstitute.org/annotation/genome/fusarium_group/MultiDownloads.html) using Bowtie v0.12.5 (http://bowtie-bio.sourceforge.net/index.shtml) [46] and TopHat v2.0.0 (http://tophat.cbcb.umd.edu/) [45, 47] with default settings. Cufflinks v0.9.3 (http://cufflinks.cbcb.umd.edu/; [48]) was used to calculate transcript abundance based on fragments per kilobase of transcript per million fragments mapped (FPKM) using all parameters on default settings. The transcript was considered as expressed when the FPKM value was greater than 0.1 and the lower boundary for FPKM value was greater than zero at 95% confidence interval.

Once the transcript abundance was calculated for individual sample files using Cufflinks, the output files were further merged pairwise for each comparison (*in vitro* comparison between two populations, *in planta* comparison between two populations and *in planta* versus *in vitro* for each population) using Cufflinks utility program-Cuffmerge [45]. The pairwise comparisons of gene expression profiles between the two populations were done using the Cuffdiff program of the Cufflinks version 1.3.0 [48]. The genes were considered significantly differentially expressed if Log2 FPKM (fold change) was ≥1.0 and false discovery rate (FDR, the adjusted P value) was <0.01. The q-value which is a positive FDR analogue of the p-value was set to <0.01 [49].

In order to visualize the expression data from all samples into two dimensions, principal component analysis (PCA) was performed using JMP Genomics v 6.0 (SAS Institute Inc., Cary, NC) for all genes (except novel transcripts). The expression data were transformed using mean normalization prior to PCA. The expression data of individual conditions were divided by their mean values across all treatment conditions to neutralize influence of hidden factors.

### Functional categorization of differentially expressed genes (DEGs)

The differentially expressed genes (DEGs) were functionally categorized online for all pairwise comparisons according to the Munich Information Center for Protein Sequences (MIPS) functional catalogue [50]. The functional categories and subcategories were regarded as enriched in the genome if an enrichment P- and FDR- value was below <0.05. The KEGG (Kyoto Encyclopedia of Genes and Genomes) pathway analyses were performed using interface on Blast2GO (Blast2GO v2.6.0, http://www.blast2go.com/b2ghome) for all DEGs to identify gene enrichment on a specific pathway.

#### Quantitative real time-PCR (RT-PCR) analysis

RT-PCR analysis was done according to the method described by Leng et al. [51]. Briefly, total RNA was extracted from each of the samples as collected for RNA-seq using the SV Total RNA Isolation Kit (Promega, Madison, WI) and purified by treatment with DNase I (NEB, Ipswich, MA) according to the manufacturers’ manuals. The reverse transcription reaction was performed on 2μg of total RNA using the SMART MMLV Reverse Transcriptase (Takara, Mountain View, CA). cDNA was diluted 20 times and used as template for quantitative RT-PCR, which was performed with the CFX96 real time PCR system (Bio-Rad, Hercules, California). Primers used for RT-PCR were listed in S5 Table. For each cDNA sample, three replications were performed. Each reaction mixture (20 μl) contained 5μl of cDNA template, 10μl of SYBR^®^ Green PCR Master Mix (Applied Biosystems, Foster, CA) and 0.3μl of each primer (10 μM). Relative expression levels of genes were normalized using the beta-tubulin gene as internal control, and were calculated as the fold change by comparison between *in planta* and *in vitro* (axenic culture) samples.

### Statistical analyses

Significance of differences between 3ADON and 15ADON populations in DON accumulation on each wheat genotype, number of total reads, and total expressed genes were analyzed using proc test in SAS (version 9.4; SAS Institute, Cary, NC). The standard deviation and standard error were calculated using Microsoft Excel 2013. The area-proportional Venn diagrams were created using the online tool (http://bioinforx.com/free/bxarrays/overlap.php) and Microsoft PowerPoint 2013.

### Sequence data accessibility

The sequence data have been uploaded to the NCBI Short Read Archive [GEO# GSE83735]

## Results

### Aggressiveness and DON accumulation of the two fungal populations on spring wheat genotypes

**Fig 1:**
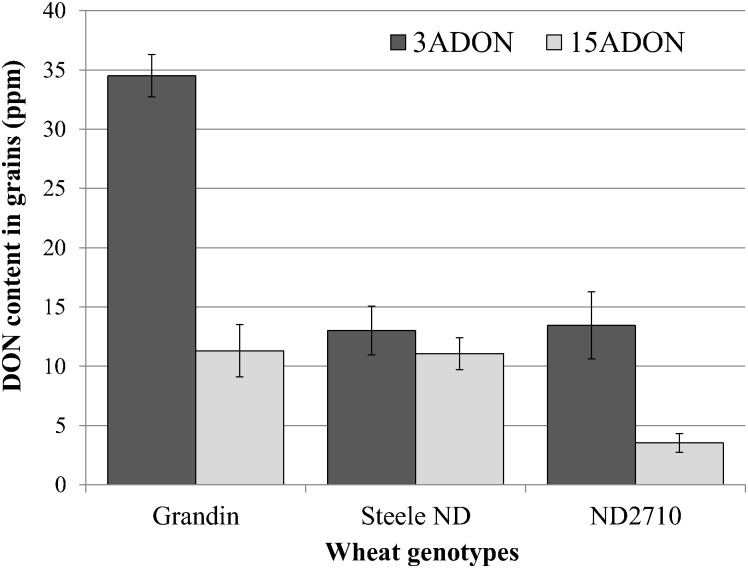
Total amount of deoxynivalenol (DON) accumulated on wheat grains. DON was obtained from spikes of three wheat genotypes (Grandin, Steele-ND, and ND 2710) after inoculations with 3ADON and 15ADON isolates, respectively. The 3ADON population accumulated a significantly higher DON on Grandin (p < .0001) and ND 2710 (p = 0.0034) than the 15ADON population. However, no significant difference (p = 0.4366) in DON accumulation on Steele ND was found between the two populations.

No significant difference in aggressiveness was observed between the 3ADON and 15ADON populations on the three wheat genotypes based on disease severity (DS). The average DS on the resistant genotype (ND 2710) was 29.6±7.2% and 27.9±10.1% for the 3ADON and 15ADON populations, respectively. On the susceptible cultivar (Grandin), the average DS was 65.3±11.4% for the 3ADON population and 56.8±16.3% for the 15ADON population. On the moderately resistant cultivar (Steele-ND), the average DS was 52.0±9.5% for the 3ADON population and 54.2±7.1% for the 15ADON population. However, the 3ADON population accumulated a significant higher DON content on Grandin (t = 8.1, p< 0.0001) and ND 2710 (t = 3.4, p = 0.0034) than the 15ADON population, although the difference was not significant on Steele-ND (t = 0.8, p = 0.44) (Fig 1).

### General features of *in vitro* and *in planta* transcriptomes of 3ADON and 15ADON populations

A total of 559,577,636 sequence reads totaling 27.97 gigabase-pair (Gbp) were generated from the 4 *in vitro* and 12 *in planta* RNA samples (Table 2). The numbers of sequence reads from the *in-vitro* RNA samples ranged from 34.8 to 40.9 million for the 3ADON population, and 32.2 to 34.5 million for the 15ADON population. The sequence reads from the *in planta* samples ranged from 26.4 to 49.5 million for the 3ADON population, and 27.8 to 39.1 million for the 15ADON population. No significant differences in a total number of sequence reads were observed between the two populations (t = 0.99, p = 0.34).

**Table 2:** Summary of sequence reads (in millions) from 16 RNA samples.

Summary^a^	*in vitro*				*In planta*											
					48 HAI				96 HAI				144 HAI			
	3A-R1	3A-R2	15A-R1	15A-R2	3A-R1	3A-R2	15A-R1	15A-R2	3A-R1	3A-R2	15A-R1	15A-R2	3A-R1	3A-R2	15A-R1	15A-R2
Total reads	40.92	34.87	34.58	32.23	34.42	30.27	27.81	34.50	33.43	26.48	39.18	36.94	49.56	41.21	29.17	34.01
Mapped reads	33.71	28.53	27.71	25.99	1.89	1.60	1.98	2.29	2.63	3.14	2.85	2.80	6.57	4.77	2.38	2.20
	82.4%	81.8%	80.1%	80.7%	5.5%	5.3%	7.1%	6.6%	7.9%	11.8%	7.3%	7.6%	13.3%	11.6%	8.2%	6.5%
Unique match	33.43	28.21	27.34	25.71	1.82	1.55	1.90	2.21	2.53	3.04	2.68	2.64	6.21	4.64	2.28	2.10
	81.7%	80.9%	79.1%	79.8%	5.3%	5.1%	6.8%	6.4%	7.6%	11.5%	6.9%	7.2%	12.5%	11.3%	7.8%	6.2%
Multi-position match	0.28	0.32	0.37	0.28	0.07	0.05	0.08	0.08	0.10	0.09	0.17	0.16	0.36	0.13	0.10	0.10
	0.68%	0.92%	1.07%	0.86%	0.22%	0.16%	0.30%	0.24%	0.31%	0.35%	0.42%	0.44%	0.73%	0.31%	0.35%	0.28%
Unmapped	7.21	6.33	6.87	6.24	32.53	28.68	25.83	32.21	30.80	23.35	36.33	34.14	42.99	36.45	26.78	31.81
	17.6%	18.2%	19.9%	19.4%	94.5%	94.7%	92.9%	93.4%	92.1%	88.2%	92.7%	92.4%	86.7%	88.4%	91.8%	93.5%

^a^Sequence reads from each RNA sample were mapped to the reference genome of *F*. *graminearum* (PH-1) using Bowtie v0.12.5 [46] and TopHat v2.0.0 [47]. HAI: hours after inoculation; 3A: 3ADON (3-acetyl-deoxynivalenol)-type population; 15A: 15ADON (15-acetyl-deoxynivalenol)-type population; R: Replicate.

**Fig 2:**
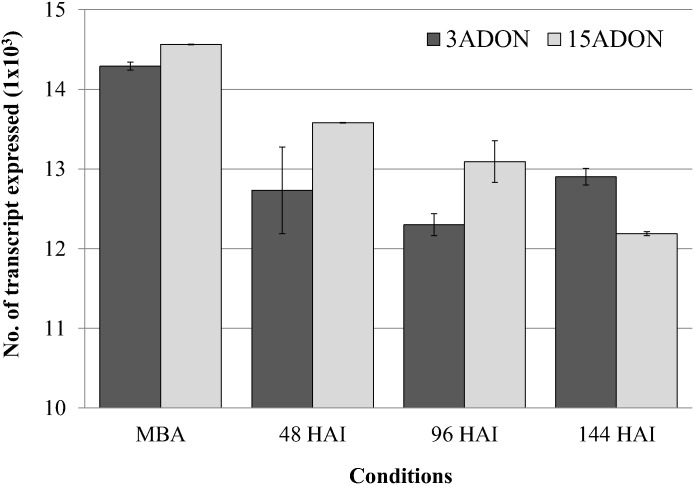
Total numbers of transcripts expressed by the 3ADON and 15ADON populations *in vitro* and *in planta*. Transcript-fragment reads from each sample were mapped to the reference genome (*F*. *graminearum* PH-1) using Bowtie version 0.12.5 [46] and TopHat version 2.0.0 [47]. Fragments per kilobase of transcript per million fragments mapped (FPKM) were calculated by Cufflinks version 0.9.3 [48]. HAI: hours after inoculation, 3ADON: the 3ADON population producing 3-acetyl-deoxynivalenol and DON, 15ADON: the 15ADON population producing 15-acetyl-deoxynivalenol and DON, vertical bar represents standard error of means between replications, MBA: mung bean agar (*in vitro*).

Of the sequence reads from *in vitro* samples, 80.1–82.4% were aligned to the reference genome of *F*. *graminearum* (PH-1), and 79.1–81.7% of the reads had a single match to the reference genome while 1.07% of the reads aligned to multiple genomic locations within the reference genome. Of the sequence reads from *in planta* samples, only 5.3–13.3% mapped to the reference genome, with unique matches accounting for 5.1–12.5% of total reads (Table 2). The number of expressed transcripts ranged from 14,242 to 14,564 *in vitro*, and 12,163 to 13,586 *in planta* (Fig 2).

**Fig 3:**
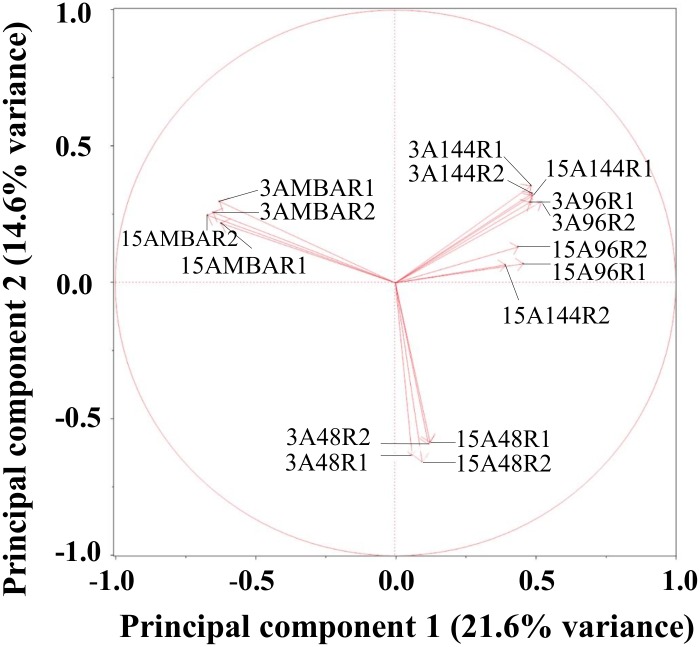
The principal component analysis of transcriptomes from 16 samples. The principal component 1 (*PC1*) describes 21.6% variance, and differentiate *in vitro* samples from *in planta* samples, while principal component 2 (*PC2*) describes 14.6% variation and differentiate early infection stage (48 HAI) from the late infection stages (96 HAI and 144 HAI). Replications from each condition were clustered together.

The principal component analysis (PCA) of transcript abundance, measured as the FPKM (fragments per kilobase of transcript per million fragments mapped) values, indicated that the RNA-seq data were very similar between the two biological replicates of each treatment (Fig 3). The principal component 1 (PC1) accounted for 21.6% variation, and clearly distinguished the *in vitro* samples from the *in planta* samples. Principal component 2 (PC2) describes 14.6% variation and differentiates the expression pattern in the early infection stage (48 HAI) from those in the late infection stages (96 and 144 HAI).

### Comparison of transcriptomes between 3ADON and 15ADON populations under *in vitro* conditions

Under *in vitro* growth conditions, 479 and 801 genes were up- and down-regulated, respectively, in the 3ADON population as compared to the 15ADON population. Among them, 454 up-regulated and 723 down-regulated genes were identified in Munich Information Center for Protein Sequences (MIPS) database using functional catalogue (FunCat) analysis (Table 3, S1 Table).

**Table 3:** Pair-wise comparison of gene expression profiles between 3ADON and 15ADON populations.

Comparison^a^	significantly up-regulated genes^b^	significantly down-regulated genes
**A. *In vitro* only**		
3ADON-vs-15ADON	479 (454)	801 (723)
**B. *In-planta* only**		
48 HAI: 3ADON-vs-15ADON	185 (177)	292 (285)
96 HAI: 3ADON-vs-15ADON	89 (85)	362 (339)
144 HAI: 3ADON-vs-15ADON	62 (59)	241 (228)
**C. *In planta* versus *in vitro***		
3ADON: 48 HAI-vs-*in vitro*	2159	1631
3ADON: 96 HAI-vs-*in vitro*	1981	2694
3ADON: 144 HAI-vs-*in vitro*	2095	2632
15ADON: 48 HAI-vs-*in vitro*	2415	1510
15ADON: 96 HAI-vs-*in vitro*	2059	1975
15ADON: 144 HAI-vs-*in vitro*	1777	2087

^a^Number of differentially expressed genes in each population were identified for three comparisons. A. comparisons under *in vitro* growth conditions; B. comparisons under *in planta* conditions at three time points after inoculation; C. comparisons between *in planta* and *in vitro* conditions for each of the 3ADON and 15ADON populations.

^b^Number of differentially expressed genes were calculated using Cuffdiff within Cufflinks v1.3.0 [48]. Genes were considered significantly up-regulated or down-regulated if FPKM (fragments per kilobase of transcript per million fragments mapped) log2 (fold change) value was greater than one at the false discovery rate (q) of 1% (<0.01). The numbers in parentheses indicate the numbers of genes found in MIPS functional catalogue [50]. HAI, hours after inoculation; 3ADON, 3-acetyl-deoxynivalenol; 15ADON, 15-acetyl-deoxynivalenol.

Among the 454 up-regulated genes found in FunCat, 276 were under ‘unclassified proteins’ and were non-significant (p = 0.977, S1 Table). The significantly enriched categories were those for metabolism (p = 1.8×10^−09^), cellular transport, transport facilities and transport routes (p = 0.033), and interaction with the environment (p = 0.0272). Within the ‘metabolism’ category, genes involved in polysaccharide metabolism (p = 8.9×10^−18^), C-compound and carbohydrate (p = 2.4×10^−16^) metabolism, extracellular (p = 5.0×10^−05^) and secondary metabolism (p = 0.0108), lipid, fatty acid, and isoprenoid metabolism (p = 0.011) as well as genes under sub-category of polysaccharide and amino acid degradation were highly significantly enriched (S1 Table). The same was true for the genes involved in non-vesicular cellular import, carbohydrate transport, heavy metal ion transport (Cu^+^, Fe^3+^), cation transport (H^+^, Na^+^, K^+^, Ca^2+^, NH_4_^+^), metabolism and FAD/FMN binding related genes.

Among the 723 down-regulated genes, 81% (586) were those encoding hypothetical proteins without any functional annotations. Of the genes with functional annotations, those for metabolism of many compounds such as melanin, membrane lipid, sugar alcohols, sesquiterpenes, glycolipid, alanine, primary metabolic sugar derivatives, and secondary metabolites, and those involved in NAD/NADP binding, and virulence factors were significantly enriched (P<0.05).

**Fig 4:**
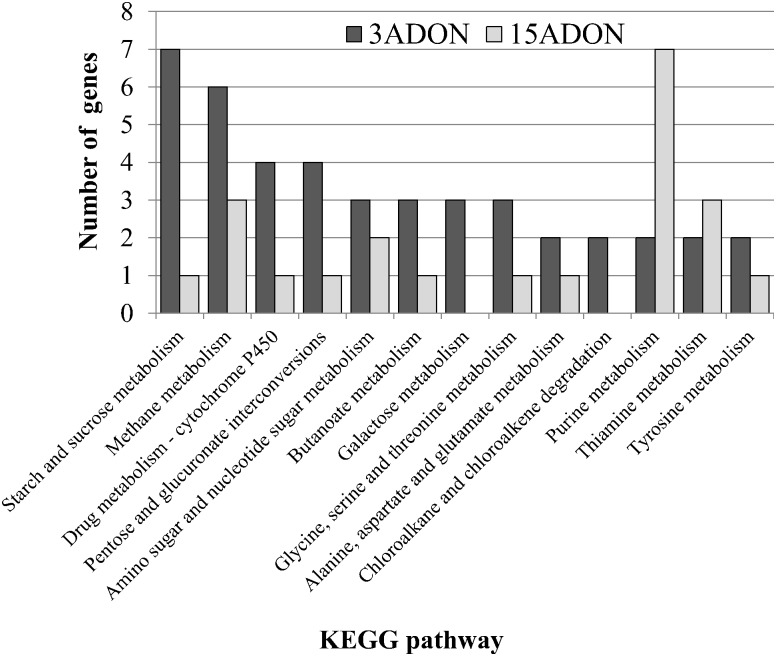
Kyoto Encyclopedia of Genes and Genomes (KEGG) pathway analyses of *in vitro* up-regulated genes. Values with log2 fold change >1 and false discovery rate (<0.01) were considered as differentially expressed. Only pathways having at least two genes up-regulated on either of the population are shown. Detailed information on KEGG pathway analysis is given in S1 Table.

KEGG pathway analysis of differentially expressed *in vitro* genes indicated that majority of up-regulated genes in the 3ADON population were involved in metabolism of starch, sucrose, methane, drugs, and in inter-conversions of pentose and glucuronate, while the down-regulated genes were those for purine, and thiamine metabolism (Fig 4, S1 Table).

### Comparisons of *in planta* and *in vitro* transcriptomes in 3ADON and 15ADON populations

**Fig 5:**
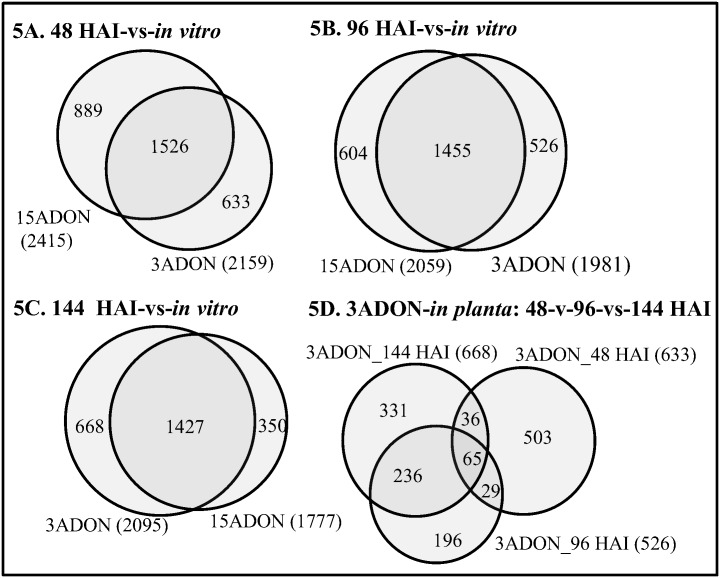
Venn diagram comparing exclusively up-regulated genes in *in planta* vs *in vitro* in the 3ADON and 15ADON populations. Samples were taken at 48 (A), 96 (B), 144 (C) hours after inoculation (HAI). D). Comparison of genes up-regulated within 3ADON population. 3ADON: population producing 3-acetyl-deoxynivalenol and DON, 15ADON: population producing 15-acetyl-deoxynivalenol and DON. Information of all genes on each Venn diagram and their functional annotation are given on S2 and S3 Tables.

A total of 2,159, 1,981 and 2,095 genes in the 3ADON population, and 2,415, 2,059 and 1,777 genes in the 15ADON population were up-regulated *in planta* when compared with their *in vitro* expressions at 48 HAI, 96 HAI, and 144 HAI, respectively (Fig 5A–5C; Table 3). More unique genes were expressed during early infection establishment, but the number of common genes was higher during disease spread even for the 3ADON population (Fig 5D, S3 Table).

**Fig 6:**
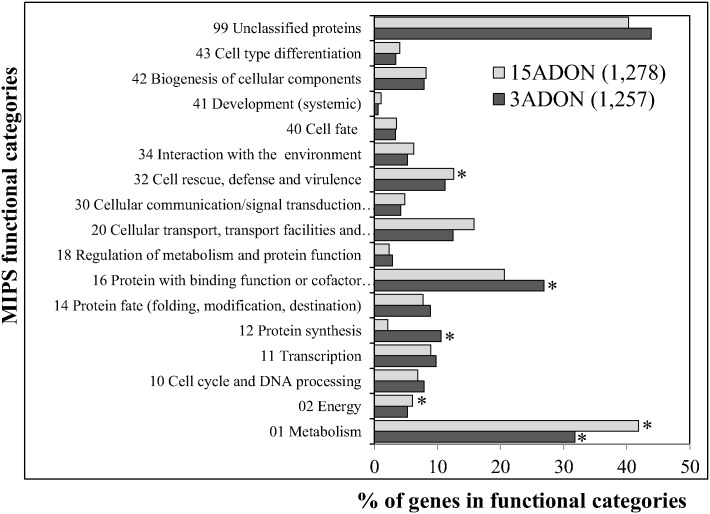
Functional analysis of *in planta* up-regulated genes in the 3ADON and 15ADON populations. The uniquely upregulated genes either in the 3ADON or the 15ADON population (all time points combined) were used for functional categorization. Total numbers of gene found in MIPS catalogue are listed in parenthesis. The functional categories in which members are significantly enriched compared with the whole genome are marked with asterisks (p < 0.05, FDR < 0.05). Information of these genes and their functional annotation are given on S4 Table.

In order to identify uniquely up-regulated genes and their functional categorization in each population, the genes specifically up-regulated in the 3ADON and 15ADON populations at each time point (633+526+688 in 3ADON; 889+604+350 in 15ADON) were summed up and duplicate genes among three time points were discarded. This gave a total of 1396 unique genes in the 3ADON population, and 1398 unique genes in the 15ADON population, respectively. Of these *in planta* up-regulated unique genes, 1257 from 3ADON population and 1278 from 15ADON population were identified in Munich Information Centre for Protein Sequences (MIPS) functional catalogue database [50]. The highest number of genes from both 3ADON (552/1257, 43.9%) and 15ADON (516/1278, 40.3%) populations belonged to ‘unclassified proteins’ category (Fig 6, S4 Table). In the 3ADON population, the up-regulated genes with functional annotations were significantly enriched in the following categories: ‘metabolism’ (400 genes, p = 0), ‘protein synthesis’ (134 genes, p = 1.2×10^−38^), and ‘protein with binding function or cofactor requirements’ (structural or catalytic) (339 genes, p = 1.7×10^−5^). In the 15ADON population, the major enriched categories were ‘metabolism’ (536 genes, p = 0), ‘energy’ (77, p = 0.0017), and ‘cell rescue, defense and virulence’ (162 genes, p = 0.0014) (Fig 6, S4 Table). Also significantly enriched were the major functional sub-categories involved in nitrogen, sulfur and selenium metabolism (p = 0.0199), pentose-phosphate pathway (p = 0.0085), RNA processing (p = 0.0002), ribosome biogenesis (p = 1.8×10^−46^), ribosomal proteins (p = 6.8×10^−40^), translation (p = 2.67×10^−26^), translation initiation (p = 0.0002), translation elongation (p = 0.0012), protein (p = 0.034), nucleic acid (p = 2.2×10^−05^) and RNA binding (p = 5.1×10^−07^), oxidative stress response (p = 0.0243), detoxification by modification (p = 0.0249), and genes related to nucleolus (p = 0.0437) (S4 Table).

Further analyses of the same gene sets using the Kyoto Encyclopedia of Genes and Genomes (KEGG) pathway identified 205 genes involved in 65 metabolic pathways for the 3ADON population, and 178 genes involved in 72 metabolic pathways for the 15ADON population (Table 4, S4 Table). In the 3ADON population, more genes involved in pathways for metabolism of purine, arginine, proline and pyrimidine, citrate cycle (TCA cycle), valine, leucine and isoleucine biosynthesis, pentose phosphate pathway, carbon fixation pathways in prokaryotes or in photosynthetic organisms were up-regulated (Table 4). In contrast, in the 15ADON population, more genes involved in nitrogen metabolism, pentose and glucuronate inter-conversions, starch and sucrose metabolism, drug metabolism—cytochrome P450, N-Glycan biosynthesis, various types of N-glycan biosynthesis, benzoate degradation, chloroalkane and chloroalkene degradation, metabolism of xenobiotics by cytochrome P450 were up-regulated (Table 4, S4 Table).

**Table 4:** Kyoto Encyclopedia of Genes and Genomes (KEGG) pathway analyses of *in planta* up-regulated genes in 3ADON and 15ADON populations as compared to their *in vitro* expression.

Pathway^a^	3ADON^b^	15ADON
Purine metabolism	15	10
Arginine and proline metabolism	11	2
Glycine, serine and threonine metabolism	8	6
Pyrimidine metabolism	8	4
Citrate cycle (TCA cycle)	7	2
Valine, leucine and isoleucine biosynthesis	7	1
Methane metabolism	6	9
Glyoxylate and dicarboxylate metabolism	6	4
Cysteine and methionine metabolism	6	3
Phenylalanine, tyrosine and tryptophan biosynthesis	6	3
Pentose phosphate pathway	6	1
Alanine, aspartate and glutamate metabolism	5	2
Pantothenate and CoA biosynthesis	5	2
Carbon fixation pathways in prokaryotes	5	1
Nitrogen metabolism	4	6
One carbon pool by folate	4	3
Glycolysis / Gluconeogenesis	4	2
Carbon fixation in photosynthetic organisms	4	-
Amino sugar and nucleotide sugar metabolism	3	5
Thiamine metabolism	3	4
Valine, leucine and isoleucine degradation	3	3
Butanoate metabolism	3	2
Fructose and mannose metabolism	3	2
Glutathione metabolism	3	2
Glycerolipid metabolism	3	2
Inositol phosphate metabolism	3	2
Lysine biosynthesis	3	2
Phenylalanine metabolism	3	2
Lysine degradation	3	1
Tryptophan metabolism	3	1
Phosphatidylinositol signaling system	3	-
Pyruvate metabolism	3	-
Pentose and glucuronate interconversions	2	8
Starch and sucrose metabolism	2	6
Tyrosine metabolism	2	4
beta-Alanine metabolism	2	2
Aminobenzoate degradation	2	1
Cyanoamino acid metabolism	2	1
Oxidative phosphorylation	2	1
Riboflavin metabolism	2	1
Steroid biosynthesis	2	1
C5-Branched dibasic acid metabolism	2	-
Glucosinolate biosynthesis	2	-
Isoquinoline alkaloid biosynthesis	2	-
Tropane, piperidine and pyridine alkaloid biosynthesis	2	-
Nicotinate and nicotinamide metabolism	1	3
Propanoate metabolism	1	3
Aminoacyl-tRNA biosynthesis	1	2
Porphyrin and chlorophyll metabolism	1	2
Toluene degradation	1	2
Drug metabolism—cytochrome P450	-	5
N-Glycan biosynthesis	-	5
Various types of N-glycan biosynthesis	-	4
Benzoate degradation	-	3
Chloroalkane and chloroalkene degradation	-	3
Metabolism of xenobiotics by cytochrome P450	-	3
Arachidonic acid metabolism	-	2
Chlorocyclohexane and chlorobenzene degradation	-	2
Fluorobenzoate degradation	-	2
Galactose metabolism	-	2
Other glycan degradation	-	2
Styrene degradation	-	2

^a^Only pathways that include at least two genes up regulated in either population were listed. In 3ADON population, total 205 genes involved in 65 metabolic pathways were identified, while in 15ADON population, total 178 genes involved in 72 metabolic pathways were identified.

^b^Number of genes on specific pathway from each population.

**Fig 7:**
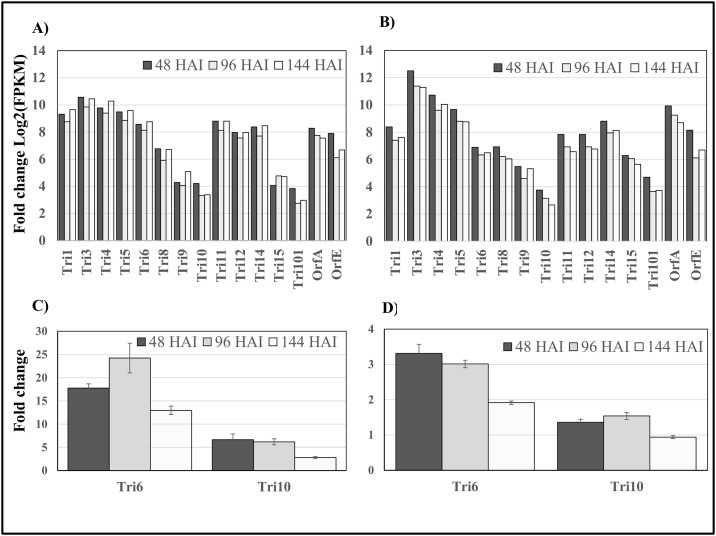
Fold change in expression of *TRI* genes during *in planta* infection compared to *in vitro* growth. A and B: Relative expression of *Tri* genes at the three infection points (48, 96, 144 HAI) compared to *in vitro* expression in 3ADON (A) and 15ADON (B) populations, respectively, based on RNA-seq analysis. Fold changes were measured using FPKM values, and were statistically significant at FDR <0.01. C and D: RT-qPCR validation of relative expression of *Tri 6* and *Tri 10 in planta* compared to *in vitro* in 3ADON (C) and 15ADON (D) populations. Relative expression levels of the *Tri* genes were normalized using the beta-tubulin gene as internal control, and were calculated as the fold change by comparison between *in planta* and *in vitro* (axenic culture) samples. Error bars represent the standard error of means.

All *TRI* genes (*Tri1*, *Tri3*, *Tri4*, *Tri5*, *Tri6*, *Tri8*, *Tri9*, *Tri10*, *Tri11*, *Tri12*, *Tri14*, and *Tri15*) involved in the biosynthesis or regulation of trichothecene production were highly up-regulated *in planta* in both populations as compared to the *in vitro* conditions (Fig 7, the values are shown in Log2 scale). The highest fold increase was observed for the *Tri3* gene in both populations followed by *Tri4*, *Tri5*, *Tri1* and others (Fig 7A and 7B). Five *Tri* genes (*Tri1*, *Tri6*, *Tri10*, *Tri11*, and *Tri12*) showed higher expression in the 3ADON population as compared to the 15ADON population. The result is consistent with quantitative RT-PCR analysis of mRNA expression for two of the *Tri* genes (*Tri6* and *Tri10*) (Fig 7C and 7D).

**Fig 8:**
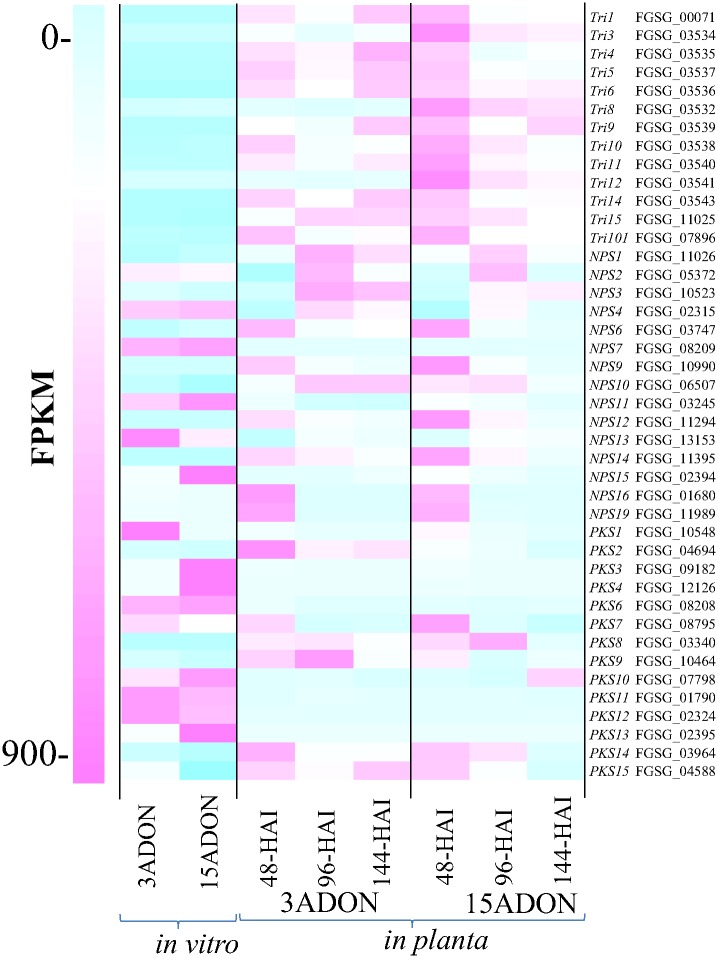
Expression of genes involved in biosynthesis of secondary metabolites. Expression level is the average of two replications.

In general, the expression levels of the genes involved in biosynthesis of trichothecenes and secondary metabolites (polyketides and non-ribosomal peptides) varied in the *in vitro* and *in planta* samples collected at the three time points. While, as stated above, most of the trichothecene biosynthesis genes were up-regulated *in planta* compared to *in vitro*, some other secondary metabolite genes (*NPS4*, *NPS7*, *NPS11*, *PKS6*, *PKS11*, and *PKS12*) had a higher expression *in vitro* than *in planta* in both populations (Fig 8). However, there was an apparent difference in *Tri* gene expression pattern between the two chemotypes. Transcripts of some *Tri* gene were high at 48 HAI, but declined at 96 HAI and recovered at 144 HAI in 3ADON population while transcripts of most *Tri* genes in 15ADON population gradually diminished during the plant infection course (Fig 8). This difference in expression pattern might be the cause for the distinct DON levels accumulated by the two populations.

Of the 65 commonly up-regulated genes within the 3ADON population across all infection time points, 61 were listed in the MIPS database (S4 Table). The majority (55.7%, 35/61) of the genes were under ‘unclassified proteins’ category. The remaining 26 genes were enriched in functions related to protein synthesis (p = 4.4×10^−6^), translation (p = 0.0002), ribosome biogenesis (p = 0.0003), translation, and amino acid metabolism (p = 0.0177), as well as metabolism of the pyruvate family (alanine, isoleucine, leucine, valine) and D-alanine, DNA processing and degradation; polyketides metabolism; peptide, antigen and GTP binding; and cation transport (H^+^, Na^+^, K^+^, Ca^2+^, NH^4+^ etc.) (S4 Table). KEGG pathway analyses identified six genes involved in five metabolic pathways, including valine, leucine and isoleucine biosynthesis (FGSG_09589, FGSG_02056); inositol phosphate metabolism (FGSG_06735); phosphatidylinositol signaling system (FGSG_06735); glycine, serine and threonine metabolism (FGSG_10211), and pantothenate and CoA biosynthesis (FGSG_02056) pathway (S4 Table).

### Comparison of transcriptomes between 3ADON and 15ADON populations *in planta*

**Fig 9:**
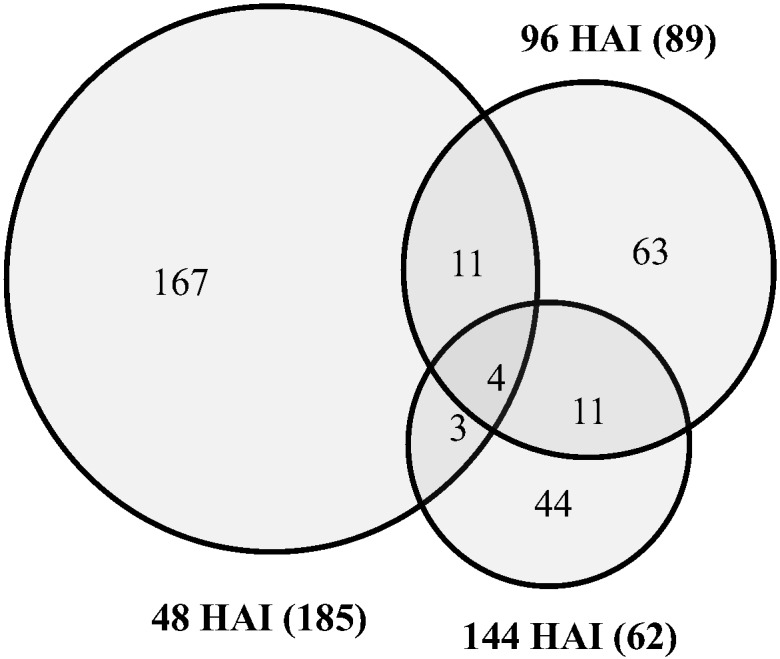
Venn diagram of exclusively up-regulated genes in 3ADON population compared to 15ADON population *in planta*. HAI: hours after inoculation, 3ADON: population producing 3-acetyl-deoxynivalenol and DON, 15ADON: population producing 15-acetyl-deoxynivalenol and DON. Information of genes corresponding to the Venn diagram are given in S2 Table.

A total of 185, 89, and 62 genes were up-regulated in the 3ADON population while 292, 361, and 241 genes were down-regulated at 48, 96 and 144 HAI, respectively, as compared to the 15ADON population (Table 3). Among them, 167, 63 and 44 genes were uniquely up-regulated at 48, 96 and 144 HAI, respectively. Only four genes (FGSG_04621, FGSG_04694, FGSG_06540, and FGSG_10632) were up-regulated at all three infection time points (Fig 9, S2 Table).

Among the 717 genes for transcription factors (TFs) identified by Ma et al. [52] in the *F*. *graminearum* genome, ten were up-regulated and 23 were down-regulated in the 3ADON population *in planta*. Of the ten up-regulated TF genes, four (FGSG_00342, FGSG_08246, FGSG_11061, FGSG_13008) were at 48 HAI, three (FGSG_01214, FGSG_04747, FGSG_09177) at 96 HAI, and three (FGSG_03881, FGSG_03695, FGSG_10277) at 144 HAI. Of the 23 down-regulated TF genes, 12 (FGSG_00196, FGSG_00725, FGSG_03292, FGSG_04083, FGSG_04293, FGSG_04747, FGSG_06436, FGSG_07116, FGSG_07482, FGSG_08617, FGSG_11271, FGSG_12134) were at 48 HAI, eight (FGSG_02874, FGSG_03399, FGSG_03649, FGSG_08064, FGSG_08954, FGSG_09368, FGSG_10030, and FGSG_13314) at 96 HAI, two (FGSG_07546, FGSG_10508) at both 96 and 144 HAI, and one (FGSG_04626) at all three infection time points.

We further examined gene families encoding host-targeted hydrolytic enzymes acting on plant proteinases, lipases, and sugar-cleaving enzymes (carbohydrate active enzymes, CAZymes), and identified six genes (FGSG_00143, FGSG_01748, FGSG_02834, FGSG_04313, FGSG_04768, FGSG_07593), three genes (FGSG_05401, FGSG_07351, FGSG_07639) and three genes (FGSG_00571, FGSG_03628, FGSG_03695) for glycoside hydrolase (GH) up-regulated at 48, 96, and 144 HAI, respectively. The genes encoding glycosyltransferases (FGSG_01882, FGSG_08902, FGSG_11341), carbohydrate esterase (FGSG_03544, FGSG_11229, FGSG_11578), and carbohydrate binding module (FGSG_11032) were up-regulated only at the early infection (48 HAI). Among 171 genes predicted to encode proteins involved in degradation of different cell components in *F*. *graminearum* [53], three [FGSG_04768 (for degradation of callose), FGSG_11032 and FGSG_11229 (for degradation of hemi-cellulose)] were up-regulated at 48 HAI, two [FGSG_07639 (for degradation of hemicellulose), FGSG_04704 (for degradation of starch)] at 96 HAI, and three [FGSG_03695, FGSG_03628 (degradation of cellulose), FGSG_00028 (degradation of protein)] at all three time points.

Functional catalogue (FunCat) analysis [50] indicated that more than 50% of differentially expressed genes identified *in planta* were for un-classified proteins, and 20.9% (37/177) and 31.2% (89/285) of the up- and down-regulated genes at 48 HAI were in the functional category ‘metabolism’ (Table 5, S2 Table). The functional category ‘cell rescue, defense and virulence’ was only significantly enriched among up-regulated genes at 96 HAI and among down-regulated genes at 48 HAI, respectively (S2 Table). Among the up-regulated genes, the functional sub-categories in glutamine degradation (p = 0.0055), C-compound and carbohydrate metabolism (p = 0.011), glutamine metabolism (p = 0.0153), arginine biosynthesis (0.0241), metabolism of urea cycle, creatine and polyamines metabolism (0.036), lipid, fatty acid and isoprenoid metabolism (0.046), assimilation of ammonia, and metabolism of the glutamate group (0.0499) were significantly enriched at 48 HAI. In addition, the genes for non-vesicular and cellular import, transport of compounds such as allantoin and allantoate, cofactor and sugar, and for post-transcriptional control were also significantly enriched (p<0.05) during the early infection stage (48 HAI). At 96 HAI, the genes for detoxification either by degradation (p = 0.0028) or by modification (p = 0.0062), and for defense related proteins (p = 0.0098) were highly enriched. At 144 HAI, the genes for secondary and polyketides metabolism, acetic acid derivatives metabolism, detoxification by modification or by degradation, and for degradation of ester compounds were abundant (P<0.05) (S2 Table).

**Table 5:** Functional annotation of *in planta* only up or down-regulated genes in 3ADON population at the three respective infection points.

Functional category	Up-regulation			Down-regulation			Whole genome^b^
	48 HAI (177)^a^	96 HAI (85)	144 HAI (59)	48 HAI (285)	96 HAI (339)	144 HAI (228)	
**01 Metabolism**	37 (20.9%)	13 (15.2%)	13 (22%)	**89** (31.2%)	63 (18.5%)	40 (17.5%)	2322 (16.7%)
**02 Energy**	3 (1.69%)	5 (5.88%)	2 (3.38%)	**18** (6.31%)	9 (2.65%)	6 (2.63%)	503 (3.63%)
**10 Cell cycle and DNA processing**	3 (1.69%)	2 (2.35%)	2 (3.38%)	9 (3.15%)	3 (0.88%)	1 (0.43%)	659 (4.76%)
**11 Transcription**	3 (1.69%)	-	1 (1.69%)	6 (2.1%)	1 (0.29%)	4 (1.75%)	718 (5.19%)
**12 Protein synthesis**	2 (1.12%)	-	1 (1.69%)	4 (1.4%)	1 (0.29%)	1 (0.43%)	370 (2.67%)
**14 Protein fate**	3 (1.69%)	1 (1.17%)	1 (1.69%)	19 (6.66%)	13 (3.83%)	5 (2.19%)	920 (6.65%)
**16 Protein with binding function**	12 (6.77%)	7 (8.23%)	5 (8.47%)	**46** (16.1%)	30 (8.84%)	19 (8.33%)	1714 (12.3%)
**18 Regulation of metabolism and protein function**	-	-	-	**11** (3.85%)	4 (1.17%)	1 (0.43%)	242 (1.75%)
**20 Cellular transport**	15 (8.47%)	10 (11.7%)	9 (15.2%)	**39** (13.6%)	41 (12%)	23 (10%)	1390 (10%)
**30 Cellular communication**	-	-	-	5 (1.75%)	4 (1.17%)	1 (0.43%)	312 (2.25%)
**32 Cell rescue, defense and virulence**	9 (5.08%)	**12** (14.1%)	5 (8.47%)	**37** (12.9%)	22 (6.48%)	16 (7.01%)	856 (6.19%)
**34 Interaction with the environment**	5 (2.82%)	5 (5.88%)	4 (6.77%)	**25** (8.77%)	19 (5.6%)	10 (4.38%)	606 (4.38%)
**36 Systemic interaction with the environment**	-	-	-	1 (0.35%)	-	1 (0.43%)	12 (0.08%)
**40 Cell fate**	1 (0.56%)	1 (1.17%)	1 (1.69%)	7 (2.45%)	4 (1.17%)	1 (0.43%)	240 (1.73%)
**42 Biogenesis of cellular components**	3 (1.69%)	1 (1.17%)	1 (1.69%)	9 (3.15%)	8 (2.35%)	4 (1.75%)	617 (4.46%)
**43 Cell type differentiation**	-	-	-	6 (2.1%)	2 (0.58%)	1 (0.43%)	273 (1.97%)
**99 Unclassified proteins**	126 (71.1%)	61 (71.7%)	38 (64.4%)	148 (51.9%)	234 (69%)	**165** (72.3%)	9004 (65.1%)

^a^Numbers in the parenthesis indicates the total number of genes found in MIPS FunCat.

^b^Numbers of genes present in each of the functional category for the whole genome were retrieved from MIPS database.

^-^indicates genes that were not detected on the specific functional category.

Numbers on bold are those significantly enriched at p = <0.05 and FDR>0.05.

A total of 50 genes in the 3ADON population showed at least five-fold expression differences at one or more time points *in planta* as compared to the 15ADON population, with 36 of them exhibiting higher expression and 24 having lower expression (Table 6). Among these 50 differentially expressed genes, 19 and 12 genes were up-regulated in the 3ADON and 15ADON populations, respectively, at least at one time point *in planta* in comparison with *in vitro* expression (Table 6).

**Table 6:** Differentially expressed *Fusarium graminearum* genes in 3ADON population showing at least 5-fold greater expression than 15ADON population *in planta* or compared to corresponding *in vitro* expression.

				Fold change	
Gene ID^a^	Condition	Gene description	Gene name	Compared to 15ADON	Compared to *in vitro*^b^
**FGSG_00002**	48HAI	Conserved hypothetical protein	..	6.5	22.7
**FGSG_00032**	48HAI	Related to non-heme chloroperoxidase	..	5.2	16.2
	96HAI	Related to non-heme chloroperoxidase	..	7.4	8.8
**FGSG_00143**	48HAI	Hypothetical protein	..	6.5	189.9
**FGSG_02321**	48HAI	Oxidoreductase that catalyses the conversion of dimeric 9-hydroxyrubrofusarin to aurofusarin	*aurO*	5.8	..
**FGSG_02324**	48HAI	Polyketide synthase that catalyse the condensation of one acetyl-coa and six malonyl-coa resulting in formation of nor-rubrofusarin	*PKS12*	35.0	..
**FGSG_02325**	48HAI	Conserved hypothetical protein		6.4	..
**FGSG_02326**	48HAI	O-methyltransferase that catalyse the methylation of nor-rubrofusarin resulting in formation of rubrofusarin	*aurJ*	10.4	..
**FGSG_02327**	48HAI	Flavin depend monooxygenase that catalyses the oxidation of rubrofusarin to 9-hydroxyrubrofusarin	*aurF*	10.2	..
**FGSG_02328**	48HAI	Laccase that catalyse the dimerization of two 9-hydroxyrubrofusarin in C7 positions	*gip1*	7.9	..
**FGSG_02329**	48HAI	Conserved hypothetical protein	..	10.0	..
**FGSG_02833**	48HAI	Probable alpha-glucoside transport protein	..	7.6	49.6
**FGSG_02966**	144 HAI	Conserved hypothetical protein	..	5.0	..
**FGSG_03335**	144 HAI	Conserved hypothetical protein	..	7.0	..
**FGSG_03336**	144 HAI	Related to integral membrane protein	..	6.6	..
**FGSG_04599**	48HAI	Related to peroxisomal short-chain alcohol dehydrogenase	..	5.1	76.7
**FGSG_04621**	48HAI	Related to monoamine oxidase N	..	8.4	4.2
	96HAI	Related to monoamine oxidase N	..	5.8	11.5
**FGSG_04694**	144 HAI	Polyketide synthase	*PKS2*	6.0	19.7
**FGSG_04717**	96HAI	Probable cytochrome P450 monooxygenase (*lova*)	..	5.2	194.7
**FGSG_04787**	144 HAI	Conserved hypothetical protein	..	5.1	..
**FGSG_05322**	48HAI	Probable fatty-acyl-coa synthase, beta subunit	..	5.1	..
**FGSG_05805**	96HAI	Related to aliphatic nitrilase	..	15.0	31.2
**FGSG_05928**	144 HAI	Conserved hypothetical protein	..	26	12.1
**FGSG_05935**	96HAI	Related to triacylglycerol lipase V precursor	..	6.6	2.1
	144 HAI	Related to triacylglycerol lipase V precursor	..	7.7	..
**FGSG_06540**	48HAI	Conserved hypothetical protein	..	17.3	9.1
	144 HAI	Conserved hypothetical protein	..	6.0	3.5
**FGSG_06580**	48HAI	Probable acetyl-coa carboxylase	..	5.7	..
**FGSG_07666**	144 HAI	Related to quinate transport protein	..	5.8	10.8
**FGSG_08076**	48HAI	Hypothetical protein	..	5.0	2.7
**FGSG_09175**	48HAI	Conserved hypothetical protein	..	6.3	..
**FGSG_10326**	48HAI	Conserved hypothetical protein	..	5.3	6.4
**FGSG_11722**	48HAI	Conserved hypothetical protein	..	6.3	4.6
**FGSG_11723**	48HAI	Conserved hypothetical protein	..	5.6	3.3
**FGSG_12049**	48HAI	Hypothetical protein	..	5.9	31.9
**FGSG_12132**	48HAI	Conserved hypothetical protein	..	5.2	2.1
**SC_3.1:373085–373840**	48HAI	..	..	5.2	..
**SC_3.2:942339–943229**	48HAI	..	..	5.2	..
**SC_3.7:2269192–2269685**	48HAI	..	..	14.1	93.5
**FGSG_02672**	48 HAI	Probable cytochrome P450 monooxygenase (*lova*)	..	-72.3	**2.5**
	96 HAI	Probable cytochrome P450 monooxygenase (*lova*)	..	-60.0	**5.3**
	144 HAI	Probable cytochrome P450 monooxygenase (*lova*)	..	-40.4	**6.5**
**FGSG_03384**	144 HAI	Probable exopolygalacturonase	..	-10.7	..
**FGSG_04008**	96 HAI	Conserved hypothetical protein	..	-13.5	..
	144 HAI	Conserved hypothetical protein	..	-13.1	..
**FGSG_04679**	144 HAI	Related to beta-mannosidase	..	-43.9	**51.0**
**FGSG_04702**	144 HAI	Related to dehydrogenase	..	-11.4	..
**FGSG_04823**	144 HAI	Hypothetical protein	..	-24.2	..
**FGSG_04892**	96 HAI	Conserved hypothetical protein	..	-12.5	..
	144 HAI	Conserved hypothetical protein	..	-41.7	..
**FGSG_07205**	96 HAI	Conserved hypothetical protein	..	-20.0	..
**FGSG_07804**	144 HAI	Hypothetical protein	..	-22.8	..
**FGSG_08960**	144 HAI	Related to kinesin light chain	..	-43.2	..
**FGSG_08961**	48 HAI	Conserved hypothetical protein	..	-12.9	..
	96 HAI	Conserved hypothetical protein	..	-59.1	..
	144 HAI	Conserved hypothetical protein	..	-87.8	..
**FGSG_09072**	96 HAI	Conserved hypothetical protein	..	-10.9	**13.5**
	144 HAI	Conserved hypothetical protein	..	-11.7	**12.3**
**FGSG_09641**	96 HAI	Conserved hypothetical protein	..	-37.8	..
	144 HAI	Conserved hypothetical protein	..	-33.5	..
**FGSG_10085**	48 HAI	Related to integral membrane protein	..	-10.7	**2.4**
	144 HAI	Related to integral membrane protein	..	-34.8	..
**FGSG_10086**	96 HAI	Conserved hypothetical protein	..	-22.1	**6.8**
	144 HAI	Conserved hypothetical protein	..	-18.1	**6.0**
**FGSG_10603**	144 HAI	Putative protein [EST hit]	..	-21.3	..
**FGSG_10636**	48 HAI	Probable IgE -dependent histamine-r-factor	..	-10.2	..
	96 HAI	Probable IgE -dependent histamine-r-factor	..	-12.7	**5.6**
	144 HAI	Probable IgE -dependent histamine-r-factor	..	-19.1	**6.3**
**FGSG_10670**	144 HAI	Probable acetylxylan esterase precursor	..	-79.0	**69.6**
**FGSG_11009**	96 HAI	Conserved hypothetical protein	..	-10.3	**117.9**
**FGSG_11449**	144 HAI	Conserved hypothetical protein	..	-13.8	**9.1**
**FGSG_13464**	48 HAI	Conserved hypothetical protein	..	-184.6	**2.8**
**FGSG_13505**	144 HAI	Conserved hypothetical protein	..	-12.7	**38.2**
**SC_3.2:5039491–5040187**	144 HAI	..	..	-61.7	**39.5**
**SC_3.2:5039551–5040054**	96 HAI	..	..	-95.8	..

^a^The differentially expressed genes were identified using Cuffdiff within Cufflinks interface [45]. Genes were considered significantly up or down-regulated in expression if the absolute value of FPKM (fragments per kilobase of transcript per million fragments mapped) Log2 (fold change) value was greater than one at the false discovery rate (q<0.01).

^b^The values indicated with bold are up-regulated fold change in 15ADON population *in planta* compared to *in vitro* expression.

^..^ indicates no information.

## Discussion

In this study, we compared relative aggressiveness and amount of DON accumulation in wheat grains between the 3ADON and 15ADON populations of *F*. *graminearum*, and also examined differences in transcriptomes between the two populations under *in vitro* and *in planta* conditions. The 3ADON population caused a higher level of disease than the 15ADON population although the difference was not significant. This result is in agreement with that of Gilbert et al [54], who compared aggressiveness of 3ADON and 15ADON isolates on the susceptible cultivar ‘Robin’ and the moderately resistant line ‘5602 HR’, and failed to find significant differences in aggressiveness. However, we found that the 3ADON population produced a significantly higher level of DON on ND 2710 and Grandin, but not on Steele ND (Fig 1). This result is consistent with previous studies [18, 55], which showed that the 3ADON population accumulated a significantly higher level of DON than the 15ADON population on both susceptible and resistant cultivars in greenhouse and field experiments.

Using microarrays, Lysøe et al. [35] studied the global gene expression pattern of *F*. *graminearum* (a 15ADON producer) during infection of the susceptible wheat cultivar ‘Bobwhite’, and showed the number of expressed genes increased from 48 hai (>4000 genes) to 96 hai (>8000), but declined after 144 hai. However, our data showed the number of expressed genes was relatively consistent for the 3ADON population at the three time points although the number of expressed genes decreased as the infection progressed for the 15ADON population (Fig 2). The discrepancy between our study and Lysøe et al. [35] could be due to different methods used for RNA sampling and expression measurements. We collected only inoculated spikelets at each infection time point for RNA extraction, while Lysøe et al. [35] harvested whole spikes for RNA isolation. The higher number of expressed genes found at a later stage for the 3ADON population may reflect its more vigorous aggressiveness and higher DON production than the 15ADON population during infection. In contrast to microarrays, the RNA-seq method potentially quantifies all of the expressed RNA from both the pathogen and the plant. We detected more than 12,100 gene transcripts at all three infection stages studied while Lysøe et al. [35] detected less. The significantly higher number of transcripts detected in our study than in Lysøe et al. [35] may be due to the more sensitive nature of RNA-seq than the microarray technique for detecting rarely expressed transcripts [41, 56].

The comparative analyses of *in vitro* transcriptomes between the 3ADON and 15ADON populations identified differentially expressed genes associated with several physiological and cellular metabolic processes. Remarkably, the genes annotated for cellular transport and transport facilities and those involved in active metabolism of internal metabolites and on nutrients uptake were significantly enriched in the 3ADON population. Seong et al. [57] identified a considerably higher number of genes (216) annotated for permeases or transporters during conidial germination (0-24h), and in fresh spores and hyphae under nutrient limiting conditions. Hallen et al. [58] analyzed changes in gene expression during perithecial development. Among 162 predicted ions transporter genes, 44 were upregulated at least two fold during sexual development. In many fungi, nutrient deficiency is prominent phenomenon during sporulation and early infection process that requires transport of various nutrients, nitrogen and carbon sources [59]. Thus, expression and enrichment of transporter genes in 3ADON population might be essential for metabolism and uptake of various carbon and nitrogen compounds during the nutrient starvation period to facilitate higher spore yield. FGSG_08403 is one of 12 highly up-regulated TFs in 3ADON population. This gene is required for perithecial development and ascospore production in *F*. *graminearum* [60]. It is not known if the higher expression of this gene in 3ADON population would be an advantage over the 15ADON population. Further investigation is needed to answer this question.

We found none of the *Tri* genes except *Tri15* and *Tri8* showed differential expression between the two populations under *in vitro* growth conditions. *Tri15* (FGSG_11025) showed a 2.8 fold increased expression while *Tri8* (FGSG_03532) had a 7.7 fold decreased expression in 3ADON population as compared to the 15ADON population (S1 Table). This result is in contrast with Walkowiak et al. [40] who found all of *Tri* genes except *Tri10* were upregulated in the 15ADON isolate (FG2) compared to the 3ADON isolate (FG1) *in vitro*. The discrepancy might be due to use of different growth conditions and *F*. *graminearum* isolates in the two studies. We used five days old mycelia grown on MBA plate while Walkowiak et al. [40] harvested mycelia from liquid culture after six hours of incubation for RNA extraction. Additionally, we used two groups of isolates for gene expression comparisons while only one pair of isolates was compared in the study of Walkowiak et al. [40].

We identified a large number of genes up-regulated *in planta* compared to *in vitro* in 3ADON and 15ADON populations (Fig 5A–5C, S2 Table). In general, all known *Tri* genes required for trichothecene biosynthesis and genes involved in secondary metabolite production were differentially expressed *in vitro* versus *in planta* in both populations (Figs 7 and 8). However, the dynamic expression changes of *Tri* genes during the three infection time points were different for the two types of isolates. Most of *Tri* genes in the 3ADON isolates showed an up-, down-, and up-regulated expression pattern at 48, 96, and 144 HAI compared to the *in vitro* conditions, whereas expression of most *Tri* genes in the 15ADON isolates was gradually decreased as the infection progressed (Figs 7 and 8). Among these genes, *Tri5* encodes trichodiene synthase involved in the first step in the trichothecene biosynthetic pathway and is required for DON synthesis [61], while *Tri8* encodes C-15 esterase or C-3 esterase, which is required for conversion of intermediate precursor into 3ADON or 15ADON product, respectively [34]. Also, four *Tri* genes, namely *Tri1*, *Tri6*, *Tri10* and *Tri11*, had at least 1.4 times greater expression in 3ADON population compared to 15ADON population across all time points (Fig 7). *Tri1*, which is located outside the *Tri*-cluster, encodes P450 oxygenase and negatively regulates the production of calonectrin [62], the intermediate precursors for 3ADON biosynthesis in *F*. *culmorum* [63]. *Tri6* is a pathway-specific transcriptional regulator in trichothecene biosynthesis pathway [30, 64], and further regulates expression of six other *Tri* genes (*Tri1*, *Tri3*, *Tri6*, *Tri7*, *Tri12* and *Tri14*) within the *Tri-*cluster and additional 192 potential genes in *F*. *graminearum* [65]. The *Tri10* gene, another regulatory factor, is required for trichothecene biosynthesis and regulation of expression of six *Tri* genes (*Tri3*, *Tri7*, *Tri8*, *Tri9*, *Tri11* and *Tri12*) in *F*. *sporotrichioides* [30]. *Tri11* encodes cytochrome P-450 monooxygenase required for hydroxylation in trichothecene biosynthesis [62]. The higher expression of these global regulating genes in 3ADON population might explain the difference in DON production between 3ADON and 15ADON populations.

Functional analysis of *in planta* up-regulated genes compared to *in vitro* between the two populations further revealed a chemotype specific gene expression pattern, and identified genes involved in distinct metabolic and molecular mechanisms. Major enriched functional categories identified were consistent with previous study [35], although some of the categories were specific to either 3ADON or 15ADON population. Genes under functional categories- nitrogen, sulfur and selenium metabolism; protein synthesis; protein with binding function or cofactor requirements and sub-categories under metabolism were highly specific and enriched in 3ADON population. Within the metabolism sub-category, the genes involved in the assimilation of ammonia, metabolism of the glutamate group, degradation, and biosynthesis of amino acids were highly enriched. It is a well-established concept that nutrients availability and their acquisition by pathogens are pre-requisites for successful colonization and fungal establishment [59]. Various nitrogen and carbon sources are required for trichothecene biosynthesis, secondary metabolite production, and virulence in many fungi including *F*. *graminearum* [66–68]. The nitrogenous compounds such as ammonia, glutamine, glutamate, and asparagine are the primary nutrient sources for many fungi including *S*. *cerevisiae*, *A*. *nidulans*, *N*. *crassa* and others. However, in case of lack or very low concentration of primary sources, fungi utilize many alternative nitrogen sources such as nitrate, nitrite, purines, amides, most amino acids and proteins after *de novo* secretion of pathway-specific catabolic enzymes and permeases [69, 70]. Thus, the expression of genes to utilize these nutrient sources during infection might have fitness advantage to the 3ADON population over the 15ADON population in survival, sporulation, aggressiveness, and higher DON accumulation.

Members of the C2H2 (Cys-Cys-His-His) zinc finger transcription factor (TF) family were expressed more abundantly in the 3ADON population than in the 15ADON population (S4 Table). Transcription factors have a diverse role in signal transduction, respiration, nitrogen utilization, peroxisome proliferation, stress tolerance, drug resistance, gluconeogenesis, sugar and amino acid metabolism and so on [71]. Among the 76 TFs with C2H2 zinc finger domains identified in *F*. *graminerum* genome [52], eight (FGSG_00764, FGSG_01298, FGSG_01350, FGSG_04288, FGSG_06701, FGSG_10350, FGSG_10470, FGSG_13964) were exclusively up-regulated in the 3ADON population (S4 Table). The function of the C2H2 zinc finger proteins in *F*. *graminearum* is unknown, but their roles have been studied in other fungi, such as calcium signaling in *Aspergillus nidulans* (*CrzA*) [72], regulation of biological processes (sexual development) (*SteA*) in *Aspergillus* [73], ustilagic acid biosynthesis (*Rua1*) in *Ustilago maydis* [74]. Of the TF encoding genes characterized by Son et al. [60] using deletion mutation, five genes (FGSG_00764 and FGSG_01298 with C2H2 zinc finger domain, FGSG_09286 and FGSG_10142 with bZIP domain, and FGSG_09871 with bromo domain) up-regulated in the 3ADON population were involved in either virulence or DON biosynthesis or both. Thus, the 3ADON population may have unique regulation of genes making it different from the 15ADON in aggressiveness and DON production as indicated by Puri and Zhong [18] and Ward et al. [10].

Our study further revealed a set of transporter-encoding genes uniquely up-regulated and enriched in the 3ADON population (S2 Table, Fig 6, Table 5). The genes involved in the transport of carbohydrate, sugars, allantoin and allantoate, vitamine/cofactor etc. and those required for host invasion and utilization of nutrient sources such as carbohydrates, proteins, lipids, and vitamins were enriched during early infection (48HAI). The genes for carbohydrate transport and cellular import were also enriched at 144 HAI. The allantoin and allantoate transport category, which is required to utilize uric acid, a host induced catabolic compound in response to pathogen infection, was specific to wheat infection under nutrient limiting condition [35]. The enrichment of these transporter genes at 48 HAI suggests their importance during early infection. The genes for cell rescue, defense and virulence were up-regulated in 3ADON population at 96 HAI. The genes for biosynthesis of secondary metabolites and detoxification of anti-microbial plant metabolites were also significantly up-regulated in 3ADON population at 144 HAI. One of them is the cytochrome P450s, which is required for oxygenation during secondary metabolite production and contributes to fungal virulence via detoxification of antimicrobial plant metabolites [75].

Taken together, our results revealed a set of genes that were differentially expressed between 3ADON and 15ADON populations during *in vitro*, *in planta* or both conditions. Future functional analysis of these genes will provide insights into the mechanisms involved in higher DON production and aggressive behavior of the newly emerging 3ADON population.

## Supporting Information

S1 Table *In vitro* differently expressed genes. Contains information on differently expressed *in vitro* genes between 3ADON and 15ADON populations, their FunCat and KEEG analysis.
(XLSX)

S2 Table *In planta* differently expressed genes. Contains information on differently expressed *in planta* genes between 3ADON and 15ADON populations, their FunCat analysis, and the list of genes corresponding to Fig 9.
(XLSX)

S3 Table *In planta* vs *In vitro* differently expressed genes. Contains information on differently expressed *in planta* vs *in vitro* genes at all time points, and list of the genes corresponding to Fig 5(A)–5(D).
(XLSX)

S4 Table Unique *in planta* up-regulated genes. Contains list of unique *in planta* up-regulated genes in 3ADON and 15ADON population, their FunCat and KEGG analysis.
(XLSX)

S5 Table Primers used for quantitative real-time PCR. Contains list of primer pairs used for quantitative real-time PCR analysis (RT-qPCR).
(XLSX)
